# Exploring the Role of Free Tissue Transfers in the Preservation of Bone Length and Knee Joint Function after Lower Limb Trauma: A Retrospective Analysis

**DOI:** 10.3390/jpm14020160

**Published:** 2024-01-30

**Authors:** Natalia Ewa Krześniak, Chung-Chen Hsu, Shih-Heng Chen, Yu-Te Lin, Chih-Hung Lin, Youh-Hua Lo, Madonna Rica Anggelia, Cheng-Hung Lin

**Affiliations:** 1 Department of Plastic and Reconstructive Surgery, Chang Gung Memorial Hospital, Chang Gung Medical College and Chang Gung University, Taoyuan 333, Taiwan; nkrzesniak@cmkp.edu.pl (N.E.K.); hso369@cgmh.org.tw (C.-C.H.); shihheng@cgmh.org.tw (S.-H.C.); linut@cgmh.org.tw (Y.-T.L.); chihhung@cgmh.org.tw (C.-H.L.); mranggelia@cgmh.org.tw (M.R.A.); 2 Department of Plastic and Reconstructive Surgery, Centre of Postgraduate Medical Education, Prof. W. Orlowski Memorial Hospital, 01-813 Warsaw, Poland; 3 Department of Orthopaedic Surgery, Chang Gung Memorial Hospital, Chang Gung Medical College and Chang Gung University, Taoyuan 333, Taiwan; yocj86@cgmh.org.tw

**Keywords:** soft tissue deficiency, free tissue transfers, stump coverage, lower limb amputation, mangled extremity

## Abstract

Lower limb trauma often results in mangled extremities, and in some cases, complete amputation may be necessary. However, limiting the extent of amputation and preserving the major knee joint are crucial to enhance mobility and overall functionality. By providing painless soft tissue coverage on the stump, early prosthesis use and the initiation of physiotherapy become more feasible. Soft tissue transfers hold the potential to benefit patients in two essential aspects: first, resolving soft tissue deficiencies without causing bone shortening, and second, preparing the stump to enhance overall functionality. A retrospective study conducted at Chang Gung Memorial Hospital (2009–2016) focused on lower limb amputation patients who underwent soft tissue transfers at different time periods compared to those without stump reconstruction. Out of the 2391 cases of lower limb injuries treated operatively, 117 amputations were performed in 110 patients (44 above the knee and 73 below the knee). Among them, 12 patients received soft tissue transfers for limb salvage and soft tissue deficiency after amputations. It was observed that patients in this group were typically younger, predominantly female, had longer hospital stays, and underwent a greater number of surgical procedures (*p* < 0.05). Through the use of soft tissue transfers, successfully preserved tibial bone length and functional knee joint in selected patients was achieved. This approach effectively resolved soft tissue deficiencies following lower limb amputations, optimizing physiotherapy and facilitating functional rehabilitation.

## 1. Introduction

With the advent of contemporary reconstructive surgery, the crucial role of free tissue transfers in lower limb salvage (LLS) has been established. Nonetheless, there remains a debate regarding the potential use of soft tissue transfers in cases where limb preservation efforts are doubtful or unsuccessful. Once the inevitability of amputation is established, the treatment priority shifts toward ensuring future functionality.

During the planning phase for amputation, surgeons emphasize limiting the level of amputation and exploring possibilities for preserving the functional knee joint. It has been widely emphasized by several authors that amputations below the knee (BKAs) are considerably more beneficial for individuals than above-knee amputations (AKA) [1,2,3]. The elimination of one major joint in BKA cases, as opposed to two joints in AKA, results in more stable walking with only a limited increase in energy expenditure [1]. Furthermore, patients with BKA have a higher likelihood of returning to normal life with physical activity [4].

For a functional stump, an adequate volume of soft tissue is essential to cover the amputated bone. In cases where limb salvage is not possible, a few centimeters of bone debridement and smoothing of the edges are the most reasonable options. However, in cases of more proximal amputation, the decision is often influenced by the deficit of soft tissue, and various techniques such as myoplasty, myodesis, and specific flap procedures are employed to attenuate pressure on the distal end of the stump. However, traumatic amputations may present challenges, as muscle availability can be compromised due to ischemic necrosis soon after the injury, thereby reducing its protective value. Soft tissue deficiency poses even greater challenges for covering the bone stump. The lack of soft tissue is considered the most significant limiting factor, which may lead to unfavorable, more proximal amputation, potentially sacrificing an uninjured major knee joint [5].

Since Jupiter’s pioneering report in 1982 on using “spare parts” from amputated limbs as a source of healthy tissue to cover distal stumps, various types of pedicled or free flaps have been proposed for the coverage of amputated stumps (Table 1) [1,2,3,4,5,6,7,8,9,10,11,12,13,14,15,16,17]. However, these techniques are not commonly employed in clinical practice, and their use is often overlooked, especially in traumatic events. Such neglect in treatment may lead to adverse consequences, including unnecessary harm to the patient’s mobility.

The primary research question of this retrospective study, involving patients who underwent unsuccessful limb salvage attempts or amputations following traumatic lower limb injuries, is whether free tissue transfers possess the capability to mitigate the extent of amputation, thereby preserving both bone length and the knee joint. In this study, our objective was to analyze the particular circumstances where reconstructive treatment was advised and subsequently carried out. Through the examination of these cases, our aim was to acquire insights into the effectiveness and potential advantages of reconstructive interventions in the context of unsuccessful limb salvage and amputation scenarios.

## 2. Patients and Methods

### 2.1. Inclusion Criteria

We included patients who were admitted for the treatment of traumatic injuries at the Tertiary Trauma Center of Chang Gung Memorial Hospital between January 2009 and December 2016 and subsequently underwent lower extremity amputations. Chang Gung Memorial Hospital at Linkou stands as the largest tertiary trauma center in Taiwan, consistently catering to over 30% of severely lower limb trauma patients. The primary causative factor for such cases is predominantly motorcycle accidents, often resulting in severely mangled extremities. Patients who had traumatic injuries in which the limbs were treated with or without soft tissue transfers and were ultimately salvageable were excluded from this study.

### 2.2. Injury Assessment and Diagnostic Imaging Modality

Following a traumatic accident, patients were admitted to the hospital and evaluated in accordance with trauma protocols. The decision on whether to perform primary debridement with stabilization or early amputation was made based on Trauma Unit Protocols, Mangled Extremity Severity Score (MESS), and the clinical experience of the senior surgeon after consultation with the trauma team. In almost all cases, X-ray imaging, including anterior–posterior and lateral views of the injured extremities, was performed. Some patients also underwent non-contrast CT scans or contrast CT angiography to assess the possible impact of the trauma on the blood vessels.

### 2.3. Clinical Data Analysis

Data were obtained from the centralized clinical database after receiving approval from the ethics committee for this study (IRB: No 20170171B0). The collected data included demographics, the mechanism and type of injury, the extent of the injury and imaging evaluations, general conditions during admission, history of treatment with early or delayed amputation, type of amputation with the method of stump coverage, postoperative treatment, complications, and the need for reconstruction in the immediate, delayed, or late period after treatment.

### 2.4. Multi-Disciplinary Approach to Patient Care

At Chang Gung Memorial Hospital, a coordinated, multi-disciplinary approach to severely traumatized patients was established in 1989, known as the trauma team. After initial assessment, management, and stabilization by the trauma team in our emergency service, each patient is then evaluated by the Orthopedic Unit for any bone injuries and the Plastic Surgery Unit for any revascularization or soft tissue management required. Notably, the field of the orthoplastic approach to lower extremity reconstruction has emerged and is experiencing dynamic growth, with a primary focus on assisting patients with traumatic injuries. This multi-disciplinary approach enables us to select the optimal treatment option for each individual case and extend the reconstructive possibilities beyond the scope of one specialty.

### 2.5. Statistical Analysis

Statistical analysis of the data was performed using SPSS (IBM SPSS Statistics for Macintosh Version 20.0. Armonk, NY, USA: IBM Corp). Nonparametric statistical analysis tools, including Fisher exact tests and Mann–Whitney tests, were chosen for comparative evaluations. The analysis aimed to identify factors that distinguished patients treated with conventional methods from those who received reconstructive procedures.

## 3. Results

In total, 117 amputations were performed in 110 patients out of the group of 2391 lower limb traumatic injuries seen over an 8-year period (2009–2016) at Chang Gung Memorial Hospital. This represents a rate of 4.89% of lower limb traumatic injuries leading to amputation during the study period. Among these amputations, 37.6% (44 amputations in 42 patients) were AKA, while 62.4% (73 amputations in 68 patients) were BKA.

The patients were divided into two groups: the Reconstructive Surgery (RS) group consisted of 12 patients who received 17 reconstructive procedures, first for limb salvage and subsequently (after inevitable amputation) to address tissue deficiencies. Reconstructive treatment was chosen in this group based on patient expectations of no further limb shortening and reconstruction with free tissue transfers, regional flaps, or spare parts surgery to cover the stump. On the other hand, the Non-Reconstructed (NR) group included 98 patients who were treated with conventional methods for stump cover.

The two groups exhibited a significant difference in age, with the RS group having an average age of 35.2 years, while the NR cases had an average age of 55.5 years (*p* < 0.05). Furthermore, the sex distribution in the RS group was equal, with six females and six males (1:1 ratio). In contrast, the NR group had a majority of male cases (73.5%) compared to female cases (26.5%), resulting in a sex ratio of 2.7:1 (*p* < 0.05) (Table 2).

Patients in both groups did not show significant differences in BMI, general health, and the presence of concomitant diseases. The MESS, which is a prognostic factor for limb salvageability in mangled extremity injuries [18], showed a statistically significant difference only in the age component between the groups. The grades of injury as measured by the AO classification [18,19] were comparable between the groups, with no significant difference observed. The number of patients with trauma in both legs was similar between the groups (Table 2). Table 2 elucidates the demographics of the amputee population, offering valuable insights into the factors influencing the likelihood of reconstruction. Notably, it underscores that reconstruction, while beneficial, is associated with an extended hospital stay and an increased number of surgeries.

The main reason for amputation in the RS group was traffic accidents, accounting for 66.6% of cases, while in the NR group, it was 71.4%. In both groups, the predominant type of injury was an IIIC tibiofibular fracture and traumatic amputation, constituting 58.2% of cases in the RS group and 72.4% in the NR group.

The concept of “life-saving amputation” is applied in cases where the preservation of the patient’s life takes precedence over salvaging the extremity. In the NR group, 10.2% of patients experienced early deaths, while there were no fatalities in the RS group. The main reasons for these early deaths were severe injuries to critical organs, such as the head and chest, leading to internal hemorrhage. In later periods, fatal events were attributed to the evolution of sepsis, systemic inflammatory response syndrome, and multi-organ failure due to the severity of the traumatic injury.

Limb salvage treatment was more frequently chosen in the RS group compared to the NR group (75% vs. 28.5%, *p* < 0.05) (Table 2). In the NR group, 66.3% of extremities underwent amputation in the immediate period (within 24 h after admission), while 33.7% were delayed. In contrast, in the RS group, 66.7% of patients received amputation in the late period (*p* < 0.05). This indicates that the decision to proceed with limb salvage was more prevalent in the RS group.

Seventeen soft tissue transfers were performed on twelve patients. Among these, eight flaps were employed during limb salvage, with regional or free tissue transfers being used. After amputation, nine flaps were used for stump reconstruction: three flaps in two patients after AKA, five flaps in four patients after BKA, and one flap after a hemipelvectomy following an above-knee amputation (Table S1).

The soft tissue reconstructions of the stump were categorized into three groups based on the time of their implementation: immediate (performed at the time of primary injury), delayed (after adequate preparation but during the same hospitalization), and late (even a few years after amputation). Only one patient underwent immediate stump reconstruction. In the delayed and late periods, six and two patients, respectively, received reconstructive procedures.

In the RS group, various types of flaps were used to cover the stump, including fillet sole flap, plantar fillet flap, anterolateral thigh (ALT) flap, and tensor fasciae latae flap, as well as deep inferior epigastric perforator (DIEP) flaps. Soft tissue reconstructions for stump coverage were successful, regardless of the timing of the procedure. All free flaps survived, but in many cases, minor revisions were required, such as debulking surgery, flap tailoring, or skin grafts to address residual wounds. Overall, the final results of all flaps (excluding the hemipelvectomy case, which required a wheelchair constantly) improved prosthesis compliance and facilitated rehabilitation.

In the anterior–posterior radiographs, we carefully assessed the length of the tibia bone removed during amputation in both the RS and NR groups. Using a computerized system with real-length parameter evaluation, the bones were measured from the proximal end to the upper line of the fracture. In the RS group, it was observed that longer bone lengths were preserved compared to the NR group (3.2 vs. 5.3 cm of removed bone). However, due to the small number of cases, this difference did not reach statistical significance (Table 3). Table 3 delineates the fracture and amputation levels in both reconstructed and non-reconstructed groups. Despite the reconstructed group presenting with a higher level of fracture, the approach taken ensures less bone shortening. This emphasizes the significance of the treatment strategy in prioritizing bone length and knee joint preservation.

Upon analyzing each case from the RS group, we found that in three out of five cases, with very short stumps, the usage of flaps allowed for knee joint preservation. Additionally, in the other two patients, the flap usage enabled bone length preservation (Table 4). These findings highlight the potential benefits of using soft tissue transfers in preserving bone length and knee joint function in certain cases.

The use of soft tissue transfers was indeed demonstrated to be beneficial for the majority of patients. However, it was observed that patients in the RS group had longer hospital stays and underwent more operative treatments compared to the NR group (*p* < 0.05). Despite the additional hospitalization and surgical interventions, the use of soft tissue transfers in the RS group offered advantages in terms of limb salvage, bone length preservation, and knee joint function, which were not achieved in the NR group. Therefore, while the RS group required more medical attention and procedures, the overall benefits of functional and cosmetic outcomes may justify the extended hospital stay and additional surgeries.

## 4. Discussion

Approximately five percent of fatal lower extremity injuries, often resulting from high-speed traffic accidents or crash injuries, ultimately lead to unavoidable amputations [19]. Soft tissue transfer has proven to be a valuable tool in limb salvage for patients after critical trauma. In some cases, if limb salvage attempts with flap reconstruction are unsuccessful, a second flap surgery may be considered to preserve bone length and the knee joint [1,2,3,4,5,6,7,8,9,10,11,12,13,14,15,16,17].

**Figure 1:**
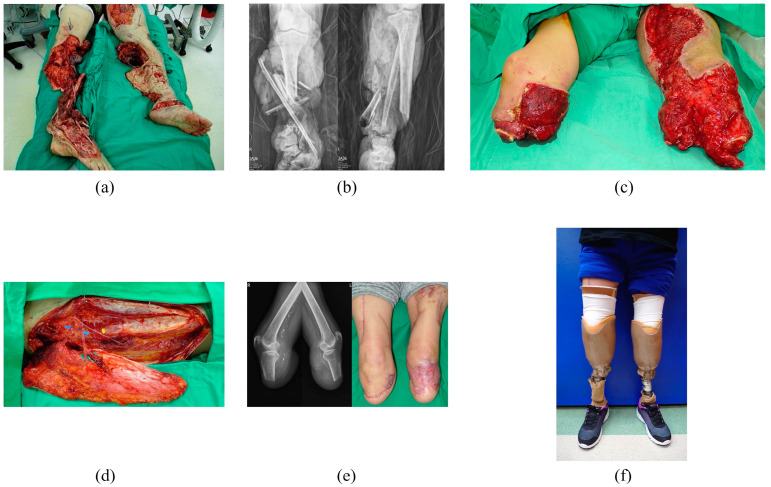
A 50-year-old woman was involved in a severe accident where she was run over by a truck, resulting in bilateral mangled lower extremities. (**a**) Due to the extent of her injuries, both legs required amputation. (**b**) Radiological examination showed severe comminution of both tibial and fibular bones, indicating the severity of the traumatic injuries. (**c**) After infection control and sequential debridements, extensive soft tissue deficiencies with exposure to both tibial stumps were noted. (**d**) Separated anterolateral thigh flap and tensor fasciae latae flap from the right thigh were used to cover both below-knee stumps to prevent further bone shortening, achieving the preservation of the knee joints. (**e**) The flap reconstruction was successful in creating functional weight-bearing stumps, providing the patient with the potential for further ambulation and mobility. (**f**) The appearance of the prosthesis six months after the injury.

**Figure 2:**
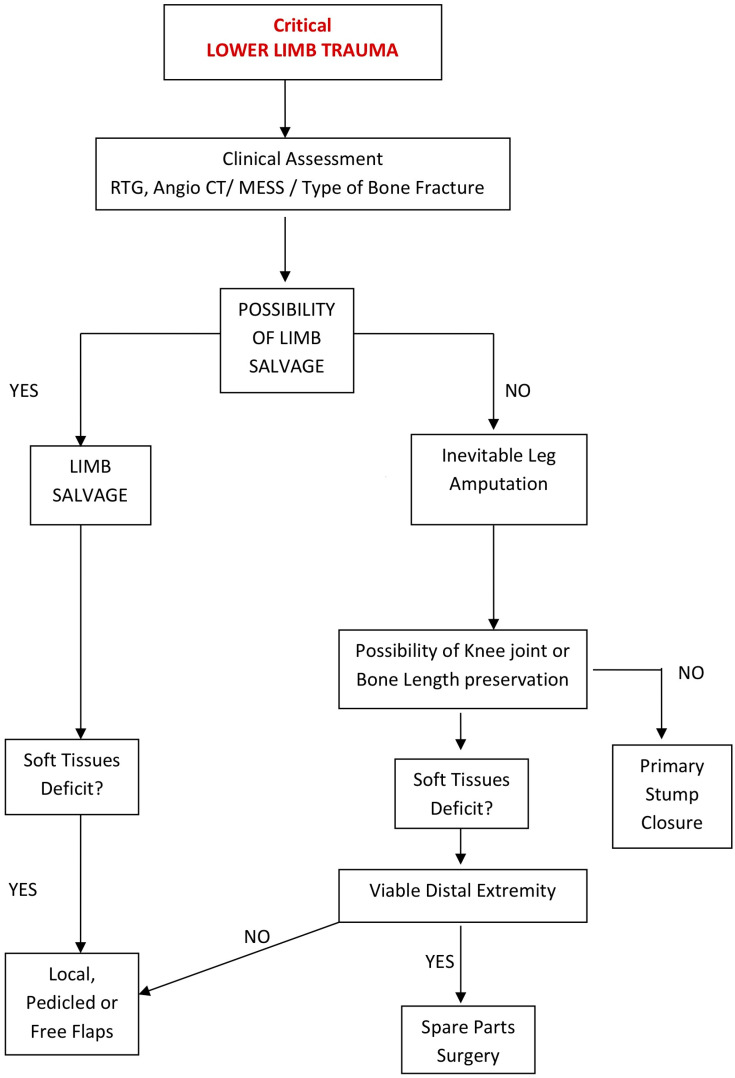
Algorithm of treatment in lower limb amputations.

The level of amputation and the method of stump coverage play a crucial role in gait rehabilitation and a patient’s ability to return to social life. While the common practice involves straightforward limb shortening by reducing bone length and using healthy soft tissues from the proximal area, this approach may result in significant bone shortening and the removal of a healthy, functional knee joint, causing irreversible harm to the patient’s future ambulation. However, the use of complex tissue transfers can be potentially beneficial in cases where there is soft tissue deficiency without the need for bone shortening, allowing for the preservation of the knee joint (Figure 1) [1,2,3,10,11,12,13,14,15,16,17,20,21,22,23,24,25]. The decision-making process for patients after traumatic amputation, based on soft tissue sufficiency, is illustrated in Figure 2.

Primary coverage of amputated stumps with “spare parts” surgery, which avoids additional donor site morbidity, is rare in clinical practice due to concerns about wound contamination, infection risk, and questionable tissue perfusion, especially in patients with severe trauma [3,10,20,21,22,23,24]. In our study, only one flap was used immediately for stump reconstruction, and the majority of reconstructions were performed later after proper debridement and wound preparation. In some cases, flaps were used in a delayed manner, around one month after the injury but still during the same hospitalization. Additionally, two flap reconstructions were performed in patients who had their stumps closed directly a few years prior and were seeking treatment due to recurrent problems known as “painful stump”. In all patients, reconstructive treatment was successful, allowed for long-term improvement, and encouraged physical activity.

The study published by Kasabian classified the time of reconstruction into four intervals: immediate, early (1–7 days), from 1 to 3 months, and above 3 months [2]. In our study, we did not perform reconstructions within the first week after the traumatic injuries. Instead, we followed a more patient-tailored approach, where most patients required a longer time, approximately one month, for repeated debridement and sometimes negative pressure wound therapy to create optimal wound conditions. This approach is in contrast to the paradigm advocated by Marko Godina, which emphasized flap coverage as early as 72 h after injury.

It is important to note that Marko Godina’s recommendations were made over 30 years ago when early wound closure was crucial to limit infection and reduce severe septic complications. However, with significant advancements in wound healing techniques, particularly the introduction of negative pressure wound therapy, early reconstruction is no longer the sole option. The use of negative pressure wound therapy allows wounds to be properly prepared over an extended period, which can lead to more favorable conditions for successful flap coverage at a later, yet optimal, time [26].

This more flexible approach to reconstruction timing that is also observed in other studies [4,8,10,16] ensures that the wound is adequately prepared, minimizing infection risk and optimizing tissue perfusion for successful flap reconstruction. By considering individual patient needs and using advanced wound healing techniques, we can achieve better outcomes and encourage long-term improvement in stump reconstruction. The focus now is on providing soft tissue reconstruction at the most appropriate moment, rather than adhering rigidly to a fixed time frame after the injury.

The statistical analysis revealed that the RS patients were significantly younger compared to the NR group, which is consistent with findings from other studies, such as the one conducted by Ghali et al. [1]. The probable explanation for this observation is that the younger population places a higher emphasis on their ability to walk and return to their regular activities, including work. As a consequence, the RS group required more operations (7.01 vs. 3.19) and longer hospital stays (52.6 vs. 29.7 days) than the NR group. The prolonged hospital stays and the need for additional procedures have been highlighted in various studies [4,8,10]. However, it is crucial to meticulously assess both the advantages and disadvantages in patients undergoing amputation.

In cases where amputation was performed at admission without attempting limb salvage or considering bone length or knee joint preservation, the treatment duration was relatively short, and some patients were discharged from the hospital within two weeks after the injury. However, this approach was typically applied to older patients, as they might have had a poorer prognosis due to their general health conditions, making amputation the more suitable choice from the beginning.

In contrast, younger patients were more likely to undergo attempts at limb salvage first, and if amputation became inevitable, preserving bone length and the knee joint was considered. In these cases, the focus was on creating a solid, functional stump that would allow for better physical activity and mobility in the future. The reconstructive treatment was viewed as an investment in improving the patient’s overall rehabilitation and long-term functionality.

By considering age and individual patient needs, the decision-making process aimed to achieve the best possible outcomes in terms of ambulation and quality of life. The goal was to provide younger patients with the opportunity for optimal functionality and independence in their daily activities, even after the traumatic event and amputation.

The literature suggests that the number of reoperations after amputation remains relatively high, with approximately half of the operated patients requiring reoperation in the first year [19,26,27]. However, in the current study, limited follow-up and a large number of patients who did not return for maintenance made it challenging to evaluate the exact number of reoperations. Nevertheless, it is highly likely that many patients in the study group required reoperation. Minor problems might have been addressed and treated in local hospitals, contributing to the difficulty in assessing the exact number of reoperations.

One of the most problematic post-amputation complications is the development of a scar on the stump, known as an adherent stump. This condition severely limits the function of the stump [6]. Skin grafts are often used to treat residual posttraumatic wounds, but they can lead to chronic issues, such as vulnerability and a tendency for non-healing wounds or recurrent ulcers on the distal stump. Such soft tissue deficits and scar contractures hinder proper weight-bearing soft tissue coverage, which affects ambulation with prostheses. Inadequate stump protection due to insufficient soft tissue volume and excessive pressure on the prosthetic device are common complaints from patients.

A variety of post-amputation complications can arise due to soft tissue insufficiency. In the early period, direct wound healing disturbances may occur, while in the long term, irritation in the stump area can impair or even prevent the proper setup of a prosthesis, reducing overall physical activity. These complications can be addressed and resolved with soft tissue transfers [4,13].

### 4.1. Clinical Implications

Preserving bone length and the knee joint after amputation have significant justifications. Lower-level amputations enable individuals to engage in more extensive physical activity with less energy expenditure and greater independence during casual and leisure activities [4,18,19,26,27,28,29,30]. Additionally, maintaining a functional knee joint allows for a more natural gait pattern, preserving lower leg kinetic moments connected to knee function. This results in less asymmetry in the pelvis position, reduces lower back pain, and lowers the risk of stump-related osteoporosis from under-use [31]. These factors contribute to better overall mobility and quality of life for lower limb amputees.

Stump length within the range of 12.5 to 17.5 cm is considered preferable for better functionality and prosthesis use. However, modern total contact sockets have made it possible to use prostheses for legs with shorter stumps, even as short as less than 8.8 cm. This advancement has allowed individuals with very short stumps to maintain functional prosthetic use [19].

Although the decision regarding the level of amputation ultimately lies with the surgeon, it is highly likely that most patients would prefer knee joint preservation over unnecessary loss. Younger, more physically active patients tend to be more adaptable to using prostheses and are often more motivated to regain their mobility and return to an active lifestyle. On the other hand, worse initial conditions in young patients can lead to a decreased chance of achieving long-term active function in the future.

Traumatic amputation of an extremity can have significant psychological effects on the patient, often resulting in symptoms of post-traumatic stress disorder. However, the psychological outcomes are inversely related to the height of amputation, meaning that higher-level amputations can lead to more severe psychological distress. Patients who undergo immediate amputation, compared to those who are involved in the decision-making process and have the opportunity to be aware of the state of their extremities, may perceive themselves as more severely traumatized in postoperative follow-up [28].

In the RS group, the ratio of early to delayed amputation was 1:2, while in the NR group, the majority of amputations were performed early, resulting in a ratio of 2:1. This difference highlights a greater emphasis on achieving functional outcomes in younger patients, which led to more delayed amputations in the RS group.

The central inquiry of this study revolves around whether free tissue transfers have the potential to mitigate the extent of amputation, preserving both bone length and the knee joint. The affirmative answer to this question is significant, as in carefully selected patients, this approach enables the preservation of bone length and the knee joint. Compilated information on the fracture level of the tibia bone after injury and stump lengths in both reconstructed and non-reconstructed groups, as presented in Table 3, clearly showed that more conservative removal of the bone was performed in the reconstructed group. While a small number of cases prevented statistical significance, there was an observed trend toward significance, with the preserved bone length being 2.1 cm longer in the reconstructed group. Without this intervention, these anatomical elements would be sacrificed, adversely affecting patients’ ability to ambulate throughout their lives.

Various soft tissue transfers for both AKA and BKA amputees were successful. Like findings from other studies, most patients required minor stump corrections to achieve final treatment closure, demonstrating the effectiveness of reconstructive techniques in improving overall functionality and prosthetic use [1,2,3,6,7,8,9,10,11].

### 4.2. Study Limitations

This study’s limitation lies in its retrospective nature; however, it provides valuable insights into clinical challenges faced by patients after traumatic amputations. Prospective studies are warranted to ascertain the optimal reconstructive treatment. Another limitation is the availability of complex reconstructive procedures, primarily accessible in hospitals with qualified microsurgeons and established microsurgical facilities. Establishing ongoing collaborations between orthopedic and plastic surgery units, along with the development of cooperative centers adopting an orthoplastic approach, holds promise for extending assistance to a larger patient population in the future [32,33].

## 5. Conclusions

Free tissue transfers remain underutilized in clinical practice for the reconstruction of amputated lower limb stumps. However, these microsurgical techniques demonstrate the potential to prevent unnecessary proximal amputations, enabling the preservation of bone length and knee joint function in carefully selected patients. Although the small sample size prevented statistical significance, there was a notable trend toward significance, with the preserved bone length being 2.1 cm longer in the reconstructed group.

## Figures and Tables

**Table 1:** Literature review for free tissue transfers in stump reconstruction (used in scenarios with soft tissue deficiencies).

	Gallico 1987 [6]	Kasabian 1991 [2]	Kasabian 1995 [7]	Küntscher 2001 [3]	Tukiainen 2002 [8]	Ghali 2005 [1]	Lu 2016 [9]	Kim 2016 [10]	Tos 2017 [11]	Current Study
Flap number										
Total	5	24	6	25	10	6	11	31	8	7
BKA	5	24	6	12	9	3	11	15	8	4
AKA	0	0	0	4	1	3	0	2	0	2
Sacral	0	0	0	9	0	0	0	Foot 14	0	1
Etiology	Trauma	Trauma	Trauma	Trauma, Tumor, Pressure	Trauma	Trauma	Trauma	Trauma DM	Trauma	Trauma
Flap Type	Mixed, Free	Mixed, Free/Pedicle	Foot fillet flaps	Fillet flap, Free/Pedicle	Free, Latissimus Dorsi	Fillet flap, Pedicle	Free Sural Neurocutaneous Perforator Flap	Free mLD +/− mSA	Fillet flap, Pedicle	Mixed, Free/ Pedicle
Time of Reconstruction	Immediate–3 Y	1 D–3 M	1–69 D	n/a	Immediate–22 Y	Delayed	Immediate	Immediate/ Delayed	Immediate/ Delayed	1 D–3 Y
Length of Hospitalization	3–8 W	51 D	24–118 D	n/a	17–36 D	28–76 D	n/a	n/a	n/a	54.9 D
Number of Operations	n/a	4–9	4–5	n/a	1–3	3–9	n/a	n/a	n/a	7
Flap Failure	0	1	0	0	0	1	0	1	0	0
Complications										
Early Anastomosis Revision	1				3					
Partial Necrosis		5	1	1	1				3	1
Neuroma		3								
Hematoma		2		1						
Thrombosis		1								
Need Surgical Revision	4		5		2		1		Need bone reunion	
Minimal Wound		2					2	6		2
Need Second flap			1							
Secondary Debulking					3					1
Stump Revision						5		5		
Follow-up	3–8 Y	12–116 M	13–116 M	14.5 M	3–42 M	9–32 M	15.2 M	14.3–17.8 M	3 Y	3.88 Y

AKA: above knee amputation, BKA: below knee amputation, D: day, DM: diabetes mellitus; mLD: muscle latissimus dorsi, mSA: muscle serratus anterior, M: month, n/a: not available, W: week, Y: year.

**Table 2:** Demographic features of patients after amputation and reconstruction and without reconstruction.

Parameters	Reconstructive Surgery (RS) (*n* = 12)	Non-Reconstructed (NR) (*n* = 98)	*p*-Value
Age (years)	35.25 ± 20.1	55.52 ± 21.4	0.03 *
Sex (M/F)	6/6	72/26	0.04 *
BMI	25.1	27.4	0.9
Both legs injured and one leg amputated	2 (16.6%)	18 (18.3%)	ns
One leg amputated Both legs amputated	9 (75%) 3 (25%)	92 (93.8%) 6 (6.1%)	0.36
Early/Delayed amputation ratio:	1:2	2:1	0.04 *
MESS	8.15 ± 2.19	8.36 ± 2.65	0.73
MESS—age	1.43 ± 0.77	1.06 ± 0.87	0.03 *
MESS—energy	3.57 ± 0.93	3.41 ± 1	0.70
MESS—ischemia	0.25 ± 0.44	0.16 ± 0.37	0.62
MESS—hemodynamic	2.14 ± 0.77	2.13 ± 0.95	0.61
Trauma Type:			
Traffic accident	8 (66.6%)	71 (72.4%)	0.67
Crash injury	3 (25.7%)	20 (20.4%)	1.0
Fall down	0	4 (4.08%)	1.0
Infection	0	3 (3.06%)	1.0
Burn	1 (8.33%)	0	1.0
Mechanism of injury (some patients have more than one injury):			
Tibia fracture:	5 (41.6%)	49 (50%)	0.68
IIIA	0	2 (2.04%)	1.0
IIIB	0	13 (13.26%)	1.0
IIIC	5 (41.6%)	34 (34.69%)	0.68
Traumatic amputation	2 (16.66%)	37 (37.75%)	1.0
Degloving	1 (8.34%)	11 (11.22%)	0.55
Femur fracture	1 (8.34%)	18 (18.36%)	0.59
Fasciitis necroticans	0	1 (1.02%)	1.0
Pelvis fracture	1(8.34%)	4 (4.08%)	0.78
Foot fracture	1 (8.34%)	35 (35.71%)	1.0
Knee dislocation	1 (8.34%)	0	1.0
Ischemia	0	2 (2.04%)	1.0
Limb salvage	9 (75%)	28 (28.5%)	0.03 *
Number of operations	7.01 ± 4.1	3.19 ± 2.81	0.001 *
Hospital stay	52.6 ± 27	29.7 ± 27	0.002 *
Died	0	10 (10.2%)	0.2

BMI: body mass index, MESS: mangled extremity severity score, ns: not significant, * statistically significant difference *p* < 0.05.

**Table 3:** The real-time measurements of the fracture level on the tibia bone after injury and stump lengths in reconstructed and non-reconstructed groups. Calculated lengths of removed bones, which must have been removed to cover soft tissue defects.

	Mean of Fracture Level (cm)	Mean of Amputation Level (cm)	Ratio (Amputation Level/Fracture Level)	The Length of Removed Bone (cm)
Whole group	19.5 ± 9.3	14.2 ± 5.2	0.73	5.3
Group without reconstruction	19.7 ± 9.8	14.4 ± 8.8	0.74	5.3
Group with reconstruction	15 ± 11.4	11.8 ± 4.5	0.79	3.2

**Table 4:** The level of the fractures, the lengths of the final stump from the selected patients with bone length, and knee joint preservation.

	Level of the Fracture	Length of the Stump (cm)	Bone Reduction (cm)	Preservation
Fillet sole flap	Double level 15 and 6 cm	6	0	Knee joint
Plantar fillet flap	26 cm	17	9	Bone length
Split ALT (RL)	14 cm	9.4	4.6	Knee joint
Split ALT/TLF (LL)	25 cm	8.5	16.5	Knee joint
ALT	Foot injury with degloving	16.7	0	Bone length

ALT: anterolateral tight flap, LL: left leg, RL: right leg, TFL: tensor fasciae latae.

## Data Availability

Data are contained within the article and supplementary materials.

## Supplementary Materials

The following supporting information can be downloaded at https://www.mdpi.com/article/10.3390/jpm14020160/s1, Table S1: The possibility of using flaps for soft tissue reconstruction in traumatic amputations and limb salvages.
