# Supplementary material for: Exploring the Role of Free Tissue Transfers in the Preservation of Bone Length and Knee Joint Function after Lower Limb Trauma: A Retrospective Analysis
Source: J Pers Med. 2024 Jan 30;14(2):160. doi: 10.3390/jpm14020160 (PMC10890581; doi:10.3390/jpm14020160)
Supplement: Supplementary file 1 [file jpm-14-00160-s001.zip › jpm-2712294-supplementary.pdf]

Table S1. The possibility of using flaps for soft tissue reconstruction in traumatic amputations and limb salvages.

**STUMP RECONSTRUCTION FLAPS**						
**No of reconstruction**	**Type of amputation**	**Time of reconstruction**	**Type of flap**	**Donor / Recipient vessel**	**Size of flap (cm)**	**Follow-up**
1)	BKA	Immediate	**FREE Fillet sole flap**	PTA /PA, VC and SV	n/a	-
2)	Hemipelvectomy	Delayed	**FREE Vastus Lateralis**	Transverse branch of LCFA / DIEA and CV	27x10	Required local plasty, and STSG
3)	BKA	Delayed	**PEDICLE Plantar fillet flap**	PTA, PTV	n/a	-
4)<br>5)	Both legs BKA	Delayed	**FREE Split ALT / TLF FLAP**	LCFA branches/ to PA and VC and to SFA and VC	n/a	STSG for residual wound
6)	AKA	Delayed	**PEDICLE: Groin flap**,	SCIA	20x6	-
7)	AKA	Delayed	**Free style island perforator flap**	Perforator of FA	15x5	-
8)	AKA	Late 3 years	**PEDICLE DIEP**	DIEA and VC	39x15	STSG for residual wound
9)	BKA	Late 3 years	**FREE ALT**	LCFA/PL and VC	25x8	debulking 1 year letter
**LIMBS SALVAGE FLAPS**						
10)	Type IIIC	Immediate	**PEDICLE Free style Fasciocutaneous rotation flap**	Local perforators	n/a	
11)	TA	Immediate	**PEDICLE GC flap**	MSA and VC	n/a	
12)	TA	Immediate	**FREE flow through ALT**	PTA and VC repair	n/a	
13)	KD	Immediate	**PEDICLE Free style Fasciocutaneous rotation flap**	Local perforators	n/a	
14)	Type IIIC	Early 5 days	**FREE ALT**	LCFA/PL and VC	n/a	
15)	Type IIIC	Delayed	**FREE Vasularized iliac osteo-cutaneous flap**	DCIA/ATA and VC	n/a	
16)<br>17)	TA, Burn	Delayed	**FREE TLF and ALT+VL for left knee defect**	LCFA/PA and VC	n/a	

BKA: Below knee amputation, AKA: Above knee amputation, , n/a: not available, VC: concomitant veins, SV: saphenous vein, PTA: Posterior Tibial Artery, PA: Popliteal Artery, LS: limb salvage, GC: gastrocnemius, LCFA:- lateral circumflex femoral artery, SCIA: superficial circumflex iliac artery, DCIA: deep circumflex iliac artery, ALT: anterolateral tight flap, FA: femoral artery, SFA: superficial femoral artery, VL: vastus lateralis, ETE: end to end, ETS: end to side, PL: Peroneal Artery, Type IIIC: Type IIIC open fracture associated with arterial injury requiring repair, TA: Traumatic Amputation, KD: knee dislocation.
